# Safety and immunogenicity of MAGE-A3 cancer immunotherapeutic with dacarbazine in patients with MAGE-A3-positive metastatic cutaneous melanoma: an open phase I/II study with a first assessment of a predictive gene signature

**DOI:** 10.1136/esmoopen-2017-000203

**Published:** 2017-11-14

**Authors:** Jean-Jacques Grob, Laurent Mortier, Lionel D’Hondt, Florent Grange, Jean Francois Baurain, Brigitte Dréno, Céleste Lebbe, Caroline Robert, Anne Dompmartin, Bart Neyns, Marc Gillet, Jamila Louahed, Silvija Jarnjak, Frédéric F Lehmann

**Affiliations:** 1 Department of Dermatology and Skin Cancers, La Timone APHM Hospital, Aix-Marseille University, Marseille, France; 2 Dermatology Department, Université Lille, INSERM U1189, Lille, France; 3 Department of Medical Oncology, Centre Hospitalier Universitaire UCL Namur (Site Godinne), Louvain, Belgium; 4 Dermatology Department, Hôpital Robert Debré, Université de Reims Champagne-Ardenne, Reims, France; 5 Department of Medical Oncology, Centre du Cancer, Cliniques Universitaires Saint-Luc, Université Catholique de Louvain, Brussels, Belgium; 6 Clinique Dermatologique, Hôpital Hôtel Dieu, Nantes Cedex, France; 7 APHP University Department of Dermatology, Saint-Louis Hospital, Paris, France; 8 Département de Médecine Oncologique, Faculté de Médecine, Gustave Roussy, Service de Dermatologie et Université Paris-Sud, Paris, France; 9 Department of Dermatology, CHU Caen, Université de Caen Basse Normandie, Caen, France; 10 Department of Medical Oncology, Universitair Ziekenhuis Brussel, Brussels, Belgium; 11 Biostatistics & Statistical Programming department, GSK, Rixensart, Belgium; 12 R&D Department, GSK, Rixensart, Belgium; 13 GSK, Rixensart, Belgium; 14 Oncology Franchise, Celyad, Mont-Saint-Guibert, Belgium

**Keywords:** immunotherapy, melanoma

## Abstract

**Background:**

We assessed safety, immunogenicity and clinical activity of recombinant MAGE-A3 antigen combined with AS15 immunostimulant (MAGE-A3 immunotherapeutic) in association with dacarbazine in patients with metastatic melanoma.

**Methods:**

In this open-label, phase I/II, uncontrolled multicentre trial conducted in Belgium and France, patients with MAGE-A3-positive melanoma received up to 24 doses of MAGE-A3 immunotherapeutic (four cycles) coadministered with eight doses of dacarbazine. Adverse events (AE) were recorded until 31 days postvaccination, and serious AEs (SAE), until 30 days following the last dose. MAGE-A3-specific antibodies were measured by ELISA. Clinical activity of MAGE-A3 immunotherapeutic was assessed in patients positive/negative for previously identified gene signature (GS) associated with clinical outcome.

**Results:**

Forty-eight patients were enrolled and treated (32 GS+, 15 GS−, 1 unknown GS status); two patients completed the study. All patients reported AEs, the most common were ‘general disorders and administration site conditions’ (94%). Treatment-related AEs were reported by 85% of patients; the most common was pain at injection site (38%). Sixteen SAEs were reported by 21% of patients; two were considered as treatment related (neutropenia and thrombocytopenia; grade 4). Postdose 4, all patients were seropositive for MAGE-A3-specific antibodies, with a geometric mean titre of 2778.7 ELISA units (EU)/mL (95% CI 1638.3 to 4712.8). One complete and three partial responses were reported (only in GS+ patients). Median overall survival was 11.4 months for GS+ and 5.3 months for GS− patients.

**Conclusion:**

Although this trial shows poor results compared with the new results with checkpoint inhibitors, it gives an interesting insight in rapidly developing fields like combinations of immunotherapy and chemotherapy, new generation vaccines and the use of gene profile as a predictive marker.

**Trial registration number:**

NCT00849875.

**Key questions**

**What is already known about this subject?**

- Treatment options for patients with metastatic melanoma include immune-checkpoint inhibitors and targeted therapies, but more specific tumour antigen-targeted cancer vaccines represent a potential therapeutic approach.
- A gene signature (GS) composed of 84 immune-related genes associated with clinical benefits of the MAGE-A3 immunotherapeutic was identified in patients with malignant metastatic melanoma or adjuvant non-small cell lung cancer.

**What does this study add?**

- This study is the first to evaluate GS profiling as a way to predict survival. The few patients who achieved an objective response under treatment with MAGE-A3 immunotherapeutic combined with dacarbazine (8.3%) were GS positive.
- Treatment with MAGE-A3 immunotherapeutic combined with dacarbazine was well tolerated, but the response rates were not superior to those observed in other melanoma studies with MAGE-A3 immunotherapeutic, and remained much lower than the rates that have been reported with checkpoint inhibitors since the time of this study.

**How might this impact on clinical practice?**

Despite poor clinical results in the context of new targeted and immune strategies, this study gives interesting information about the predictive profiling of patients which is crucial for patient selection.

## Introduction

Cutaneous melanoma is the most aggressive form of skin cancer with 55 500 deaths from malignant melanoma reported worldwide in 2012.^1^ Globally, about 132 000 people are diagnosed with melanoma every year.^2^ Patients with stage IV melanoma have a poor prognosis, with a mean survival of approximately 8 months and a 5-year survival rate of 15%–20%.^3 4^ Treatment options for patients with metastatic melanoma have changed in the last years from chemotherapy, predominantly dacarbazine to immune-checkpoint inhibitors such as anti-CTLA-4 antibody (ipilimumab), anti-programmed cell death 1 (PD-1) receptor (nivolumab and pembrolizumab) and anti-PD1 ligand (PD-L1); and targeted therapies, such as combination of MEK and BRAF inhibitors (vemurafenib and dabrafenib).^5–8^

This study was initiated before the introduction of checkpoint inhibitors and BRAF inhibitors in the daily practice for metastatic melanoma. Nevertheless, cancer vaccines targeting tumour antigens represent a potential therapeutic approach,^5 9–12^ and clinical trials testing combination of cancer vaccines with checkpoint inhibitors are currently ongoing. Similarly, chemotherapy has disappeared from the initial management of melanoma, but is actually considered as a potential synergistic agent with immunotherapy by releasing tumour antigens.

Finally, one of the biggest hurdles for any treatment is the identification of biomarkers predictive of a benefit with a given drug. Gene expression profiling of tumour samples is a powerful method for identifying gene signatures (GS). In a previous retrospective study, a GS composed of 84 immune-related genes associated with clinical benefits of the recombinant melanoma-associated antigen (MAGE)-A3 combined with AS15 immunostimulant (recMAGE-A3+ AS15, further referred to as MAGE-A3 immunotherapeutic) was identified in patients with malignant metastatic melanoma or adjuvant non-small cell lung cancer (NSCLC).^13^

For all these reasons, although this combination is no longer a first rank opportunity for patients this study evaluated the safety, immunogenicity and clinical activity of MAGE-A3 immunotherapeutic combined with dacarbazine in patients with metastatic cutaneous melanoma and evaluated a GS profiling on pretreatment tumour biopsies as a predictive marker.

## Materials and methods

### Study design

**Figure 1:**
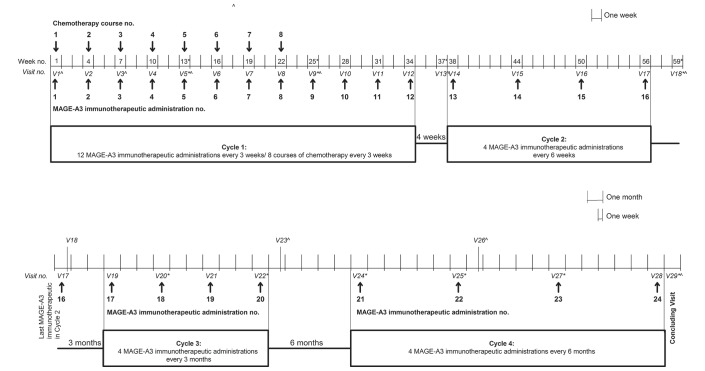
Study design.  *Tumour evaluation. ^Blood sampling for evaluation of MAGE-A3-specific antibody response. no., number; V, visit.

This study was an open-label, phase I/II, uncontrolled multicentre trial with a single group of patients conducted in 10 centres in Belgium and France between 2009 and 2012. Patients with MAGE-A3-positive metastatic melanoma received up to 24 doses of MAGE-A3 immunotherapeutic in four treatment cycles over approximately 4 years; eight doses of dacarbazine were coadministered with the first eight doses of MAGE-A3 immunotherapeutic (figure 1). A standard chemotherapy regimen consisted of dacarbazine and prophylactic antiemetic medications. The total duration of the follow-up for survival, disease progression and serious adverse events (SAE) related to the study treatment was approximately 5 years from the first treatment administration.

Treatment allocation was done by order of enrolment by the central allocation system via the internet (SBIR).

Continuation of treatment with MAGE-A3 immunotherapeutic in cycles 2, 3 and 4 was conditional upon an adequate clinical response and toxicity at the end of the previous cycle. For each patient withdrawn before visit 5, another patient was enrolled until a total of 40 patients had received ≥4 study treatment injections (visits 1–4) and completed visit 5.

All patient data were collected in electronic case report forms (eCRF). Written informed consent was obtained from each patient before the initiation of any study-specific procedures.

This study was conducted in accordance with ethical principles of Good Clinical Practice, the Declaration of Helsinki and all applicable regulatory requirements. This study is registered at ClinicalTrials.gov (NCT00849875). A protocol summary is available at http://www.gsk-clinicalstudyregister.com (GSK study ID 111714).

GVAX is a trademark of Aduro BioTech.

### Objectives

The coprimary objectives included the evaluation of (1) safety with emphasis on any possible toxic effects and (2) specific humoral and cellular immune responses induced by MAGE-A3 immunotherapeutic in association with dacarbazine.

The secondary objectives included the evaluation of the clinical activity and other indicators of safety of the study treatment.

### Study patients

Male and female patients aged ≥18 years with histologically proven, measurable MAGE-A3-positive metastatic cutaneous melanoma stage IV M1b or M1c (according to the American Joint Committee on Cancer classification),^14^ and Eastern Cooperative Oncology Group performance status of 0 or 1, who provided written informed consent prior to the study enrolment were eligible. Women of childbearing potential had to take adequate contraception for 30 days prior to administration of the study treatment, have a negative pregnancy test at the time of enrolment, and continue such precautions during the entire study treatment period and for 2 months after completion of the treatment.

Patients who received prior systemic (bio)chemotherapy or any cancer immunotherapeutic or were scheduled to receive any anticancer-specific treatments not specified in the protocol, and patients who received or planned to receive any investigational or non-registered drug or vaccine other than the study medication within the 30 days preceding the first dose of study treatment were excluded. Patients treated with systemic corticosteroids or any other immunosuppressive agents were also ineligible (details of inclusion/exclusion criteria are included in the online supplementary materials).

10.1136/esmoopen-2017-000203.supp1 Supplementary file 1

### Treatment and administration

MAGE-A3 immunotherapeutic was composed of 300 µg recMAGE-A3 antigen and a standard dose of the AS15 immunostimulant. AS15 is an immunostimulant containing 3-O-desacyl-4′-monophosphoryl lipid A (50 µg, produced by GSK), *Quillaja saponaria Molina* fraction 21 (50 µg, licensed by GSK from Antigenics, a wholly owned subsidiary of Agenus, a Delaware USA corporation) and CpG 7909 synthetic oligodeoxynucleotides containing unmethylated CpG motifs in a liposomal formulation.

Patients received 0.5 mL of MAGE-A3 immunotherapeutic by intramuscular injection in the deltoid or lateral regions of the thighs, alternately on the right and left sides. Prophylactic antiemetic medication was administered before and after each course of chemotherapy, according to standard procedures at the study site. Dacarbazine (initial dose of 1000 mg/m²) was administered every 3 weeks, with a maximum of eight courses of chemotherapy, by an intravenous injection over 1 hour (figure 1).

### Study procedures and blood sampling

At screening (up to 4 weeks before the first administration of MAGE-A3 immunotherapeutic), skin lesions were biopsied and fresh tumour samples were taken for the analysis of MAGE-A3 expression by reverse transcriptase PCR,^15^ and for the presence or absence of GS (by microarray) that may predict favourable clinical outcome identified in the phase II melanoma trial, as previously described.^13^

The full list of study procedures is included in online supplementary table S1. Blood samples (2×5 mL) for MAGE-A3-specific antibody responses were taken at predefined timepoints (figure 1).

### Safety assessment

All adverse events (AE), except autoimmune AEs, occurring within 31 days after each vaccination and SAEs occurring until 30 days following administration of the last dose of study treatment were recorded in the patient’s eCRF. Severity of AEs was assessed according to the International Common Terminology Criteria for Adverse Events (version 3.0).

All local (injection site) reactions were considered causally related to the administration of MAGE-A3 immunotherapeutic. Causality of all other AEs was assessed by the investigator.

Haematological and non-haematological toxicities considered by the investigator to be caused by the chemotherapy regimen (eg, alopecia, nausea, vomiting, neutropenia or neutropenic fever) were not reported as SAEs. The list of autoimmune diseases and other immune-mediated inflammatory disorders is included in online supplementary materials.

### Immunogenicity assessment

MAGE-A3-specific antibodies were measured by ELISA at predefined timepoints (figure 1). The ELISA assay cut-off was 27 ELISA units (EU)/mL. MAGE-A3 cellular (T cell) responses were not assessed due to the early termination of the study.

### Clinical activity assessment

Clinical activity was evaluated in the overall population, and separately in patients with GS-positive (GS+) tumours and GS-negative (GS−) tumours.

Objective tumour response was measured according to the response evaluation criteria in solid tumours (online supplementary materials).^16^

Progression-free survival (PFS), PFS after initial slow progressive disease (SPD) and overall survival (OS) were assessed (see definitions in the online supplementary materials).

### Statistical methods

The statistical analyses were performed using the Statistical Analysis Systems V.9.2 on Unix.

The target sample size of 40 patients to ensure about 20 patients in each gene profile subset was based on general experience rather than on a formal estimate or hypothesis; 30 patients were planned to be evaluated for immunogenicity.

The success criterion was the observation of MAGE-A3-specific response after the fourth dose of MAGE-A3 immunotherapeutic in at least 70% of patients.

The total treated population (TTP) included all patients who received at least one dose of MAGE-A3 immunotherapeutic. All safety analyses were performed on the TTP. The according-to-protocol population for analysis of immunogenicity included all patients who met all eligibility criteria for enrolment, did not report major protocol deviations, received at least the first four MAGE-A3 immunotherapeutic administrations concomitantly with the standard chemotherapy regimen and had a valid result for immunogenicity evaluation within 4 weeks postdose 4.

Seropositivity was defined as an antibody concentration greater than or equal to clinical cut-off value. Seropositivity rate was defined as the proportion of seropositive patients.

Humoral response to treatment was defined as an antibody concentration greater than or equal to the clinical cut-off value for patients with concentrations below the clinical cut-off value before treatment initiation; or an antibody concentration greater than or equal to twice the patient’s own baseline value for patients with concentrations above the clinical cut-off value before treatment initiation.

The antibody geometric mean concentration (GMC) was calculated by taking the antilogarithm of the mean of the log_10_ concentration transformations. Antibody concentrations below the cut-off value of the assay were given an arbitrary value of half the cut-off value for the purpose of GMC calculation. MAGE-A3-specific GMCs were calculated with 95% CIs.

The analysis of clinical activity was performed on the TTP. Objective response rate was defined as the proportion of patients whose best overall response was complete response (CR) or partial response (PR). Survival analysis was performed using the Kaplan-Meier method; two-sided 95% CIs for the median survival were computed by the Brookmeyer and Crowley method.^17^

## Results

### Study patients and treatment compliance

**Figure 2:**
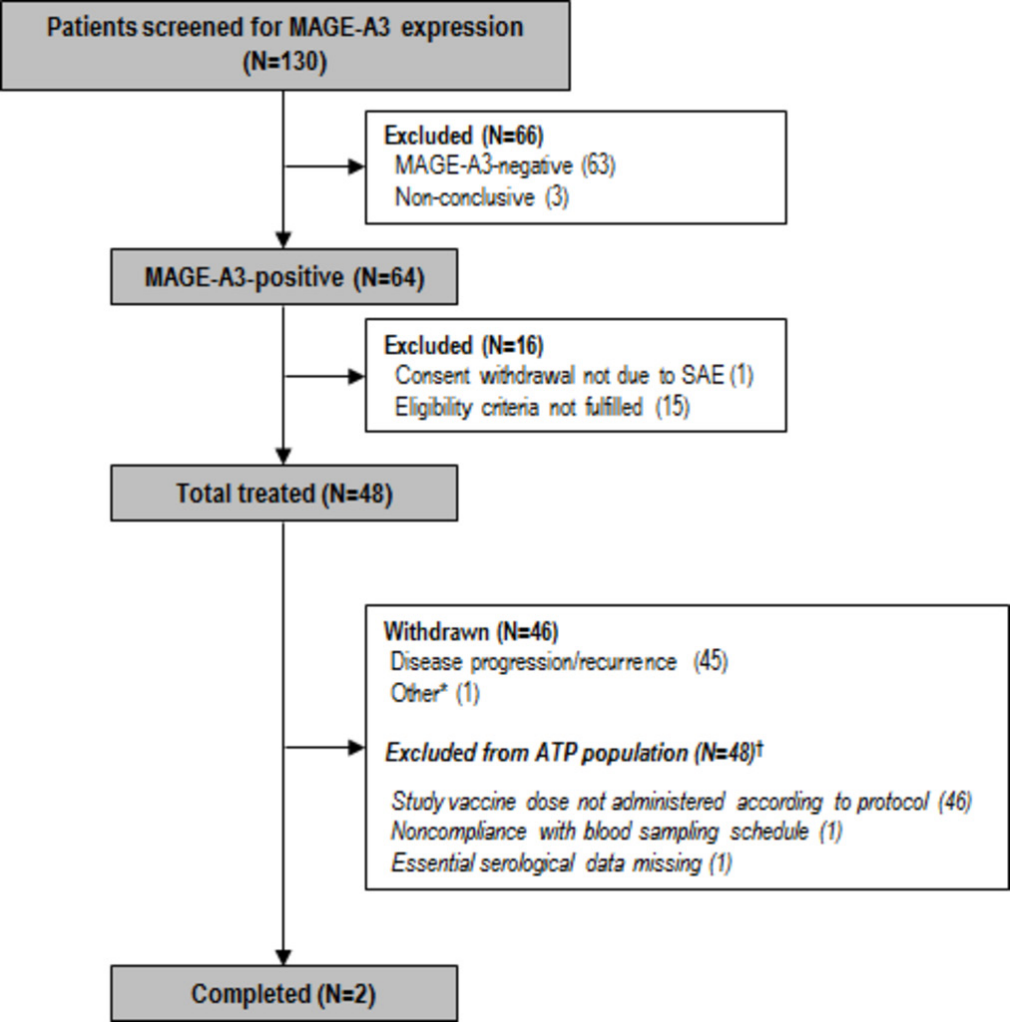
Participant flow. *Patient was withdrawn after visit 4 due to a non-AE/SAE-related reason, that is, ‘neutropenia induced delay’. †All data collected after protocol violation were eliminated from the ATP immunogenicity analyses. AE, adverse event; ATP, according to protocol; N, number of patients; SAE, serious adverse event.

A total of 130 patients were screened. Forty-eight patients were enrolled and treated (figure 2).

In total, 127 patients had valid results for MAGE-A3 expression; of those, 64 (50.4%) were MAGE-A3 positive and 63 (49.6%) were MAGE-A3 negative. Among the 64 MAGE-A3-positive patients, 36 were GS+ for their biopsy, 19 were GS− and 9 had unknown GS results.

The mean age of participants was 55.4 years (range, 22–86); 56.3% were male; all patients in the TTP (n=48) had stage IV melanoma (according to the criteria of the American Joint Committee on Cancer) and all except one received radiotherapy prior to the study entry (online supplementary table S2).

A total of 322 doses of the study treatment were administered. Among the 48 treated patients, 8 (16.7%; 7 GS+ and 1 GS−) received 12 doses of MAGE-A3 immunotherapeutic and completed cycle 1; 3 (GS+) received 16 doses and completed cycle 2; and 2 (GS+) received 24 doses and completed cycles 3 and 4.

### Safety

All patients reported at least one AE during the 31-day postadministration period, and 15 patients (31.3%) reported grade 3–4 AEs.

The most common AEs were ‘general disorders and administration site conditions’ (94.0%) and ‘gastrointestinal disorders’ (77.0%). Among ‘general disorders and administration site conditions’, the most common were asthenia (52.0%), pain at injection site (38.0%) and fever (29.0%); for two patients the reported asthenia was grade 3–4. Among ‘gastrointestinal disorders’, the most frequently observed were nausea (46.0%), constipation (29.0%) and vomiting (27.0%); one patient reported grade 3 vomiting.

Forty-one patients (85.0%) reported at least one treatment-related AE; the most common were pain at injection site (38.0%), nausea (35.0%), asthenia (33.0%), fever (23.0%) and vomiting (21.0%). Four patients (8.3%) reported six grade 3–4 treatment-related AEs. One patient reported grade 4 neutropenia and grade 4 thrombocytopenia postdose 1 that fulfilled the definition of an SAE.

Ten patients (21.0%) reported a total of 16 SAEs. Two of these SAEs (grade 4 neutropenia and grade 4 thrombocytopenia) reported by one patient were considered by the investigator to be possibly related to study treatment. Both events started 17 days postdose 1 and resolved 8 days later, before administration of dose 2.

In addition, one patient reported two events of grade 4 neutropenia (22 days postdose 1 and 18 days postdose 2). These AEs were not reported as SAEs because they were assessed by the investigator to be related to chemotherapy.

No fatal SAEs were reported.

Two patients (4.0%) reported potential immune-mediated disorders; both experienced grade 1 vitiligo that was assessed by the investigator to be potentially related to the study treatment. For one patient, vitiligo was diagnosed on the day of administration of dose 8 (visit 8). Vitiligo was not surrounding any lesion. At the time of vitiligo diagnosis, the patient had SPD with a mixed response (MR) characterised by PR of the three baseline target lesions but with occurrence of new lesions. The MR had already been reported at visit 5 (first tumour response evaluation), 3 weeks before the diagnosis of vitiligo. The patient was withdrawn after visit 9 due to disease progression. At the concluding visit (3 weeks later), the event was not resolved. The patient died in June 2010 due to disease progression. For the second patient, vitiligo was diagnosed 173 days postdose 5, which was the last dose administered to that patient (withdrawn due to disease progression at visit 6). The best overall response for this patient was progressive disease (PD). After withdrawal, the patient was first treated with ipilimumab for 3 months, and then fotemustine for the 5 following months. Vitiligo was observed on the limbs and neck, affecting 8.0% of the body surface. It was not surrounding any lesion. No skin biopsy was performed. The patient died in October 2012 due to disease progression.

### Immunogenicity

Three of 28 patients (10.7%) were seropositive for MAGE-A3-specific antibodies prior to treatment administration.

**Figure 3:**
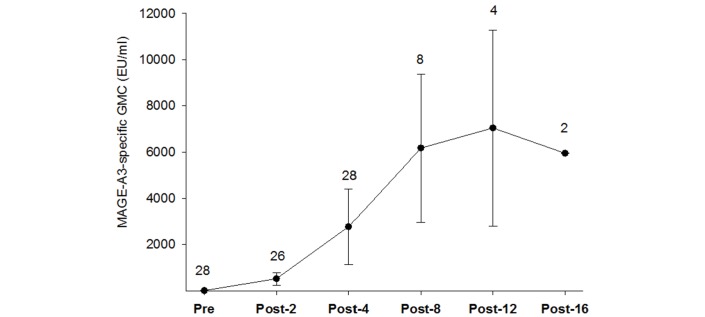
MAGE-A3-specific geometric mean concentrations (ATP population for immunogenicity). The numbers indicate the number of patients analysed at each timepoint. The error bars represent 95% CI. Due to a large CI for the last timepoint (21.3–1657225), the error bars for this timepoint are not shown. ATP, according to protocol; EU, ELISA units; GMC, geometric mean concentration; Post-2, postdose 2 (week 7); Post-4, postdose 4 (week 13); Post-8, postdose 8 (week 25); Post-12, postdose 12 (week 37); Post-16, postdose 16 (week 59); Pre, before first MAGE-A3 immunotherapeutic administration.

At 3 weeks postdose 4 (week 13), all patients were seropositive with a GMC of 2778.7 EU/mL. For the following timepoints, all remaining patients were seropositive and had responded (figure 3). The success criterion of a MAGE-A3-specific response postdose 4 observed in at least 70.0% of the patients was thus met.

### Clinical activity

#### Clinical response

An objective response (CR or PR) was reported for four patients (8.3%; all in GS+ subset) (table 1). PD was reported for 34 patients (70.8%); 22/32 patients from the GS+ subset (68.8%) and 12/15 patients from the GS− subset (80.0%). Of these, four patients (two GS+ and two GS−) presented with SPD (table 1).

**Table 1:** Best overall response by gene signature (total treated population)

	GS+ (N=32)	GS− (N=15)	Total (N=48)*
	n (%)		
Best response			
CR	1 (3.1)	0 (0.0)	1 (2.1)
PR	3 (9.4)	0 (0.0)	3 (6.3)
SD	6 (18.8)	2 (13.3)	9 (18.8)*
PD	22 (68.8)	12 (80)	34 (70.8)
NE	0 (0.0)	1 (6.7)	1 (2.1)
Best objective response	4 (12.5)	0 (0.0)	4 (8.3)
Disease control	10 (31.3)	2 (13.3)	13 (27.1)*

*One patient with SD had an unknown GS status.

CR, complete response; GS+, patients presenting gene signature; GS−, patients without gene signature; N, number of patients in the considered population; n, number (percentage) of patients in a given category; NE, non-evaluable; PD, progressive disease; PR, partial response; SD, stable disease.

Disease control, defined as CR, PR, stable disease (SD) or SD/PR, was reported for 13 patients (27.1%; 10 GS+, 2 GS−, 1 unknown). Nine patients (18.8%; 6 GS+, 2 GS−, 1 unknown) presented with SD (table 1).

MR was observed in 11 patients (22.9%).

#### OS and PFS

**Figure 4:**
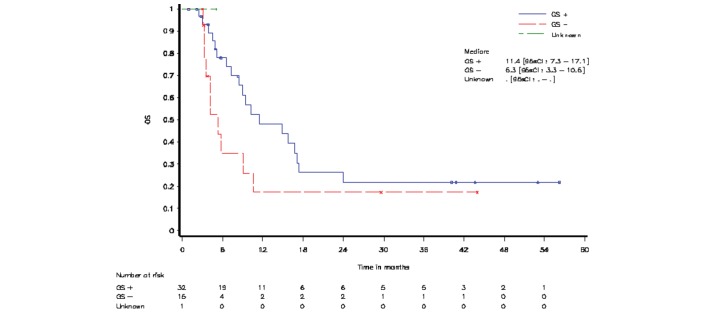
Overall survival (OS) by gene signature (total treated population). GS+, patients presenting gene signature; GS−, patients without gene signature.

Patients withdrawn regardless of the reason, who did not come for follow-up visits (30/32 in GS+ subset, 15/15 in GS− subset and  1 with an unknown GS status), and  patients still alive at the time of this analysis were censored at the date of ‘last known to be alive’. Median OS was 11.4 months for patients from the GS+ subset and 5.3 months for patients from the GS− subset (figure 4).

Median PFS and PFS after initial SPD were 2.8 months for patients from both GS subsets (online supplementary figure S1).

Median follow-up period was 40.8 months for patients from the GS+ subset and 29.6 months for patients from the GS− subset (online supplementary figure 2).

## Discussion

This study assessed combination of MAGE-A3 vaccination with chemotherapy. The results of this study indicate that it is a well-tolerated strategy. Despite poor clinical results in the context of new targeted and immune strategies for treatment of melanoma, this study gives interesting information about potential synergistic effects of different types of immunotherapies including checkpoint inhibitors, and the predictive profiling of patients which is crucial for patient selection.

The MAGE-A3 expression rate of roughly half of the patients is consistent with what was reported in a previous clinical trial in patients with stage III/IV metastatic melanoma (59%).^15^

The incidence of treatment-related AEs was high (85.0%), as reported for the same treatment combination in a phase I study in patients with NSCLC (74.0%), but grade 3 or higher treatment-related AEs were rare (8.3% in this study and 16% in the phase I NSCLC study).^18^ The most common AEs were ‘general disorders and administration site conditions’, which is in agreement with the findings of the previous phase II study in patients with melanoma who received MAGE-A3 immunotherapeutic,^15^ and with other studies with MAGE-A3 vaccination.^18–20^

In this study, 3 of 28 patients (10.7%) were seropositive for MAGE-A3-specific antibodies at baseline. Following four doses of MAGE-A3 immunotherapeutic, MAGE-A3-specific antibody response was observed in all patients, and all patients were seropositive, which is consistent with the results of previous studies with MAGE-A3 immunotherapeutic in melanoma or NSCLC.^15 18 20^

Objective response (CR or PR) was reported for four patients (one CR and three PR; 8.3%), which is similar to the objective response rates reported in previous studies of the MAGE-A3 immunotherapeutic in patients with metastatic melanoma,^15 21^ and much lower than what is observed with new checkpoint blockers immunotherapy.

In this study, the majority of patients (65.5%) were positive for the previously identified GS profiling^13^ and an objective response (one CR and three PR) was achieved in four patients who were all from the GS+ subset. However, the sample size of the present study was too small to draw any definitive conclusions about the predictive effect of the GS.

Combining cancer vaccines with chemotherapy is complicated by the fact that most chemotherapy regimens are profoundly immunosuppressive at standard doses.^22^ In a previous preclinical study, low doses of chemotherapeutic agents, such as cyclophosphamide, doxorubicin and paclitaxel, enhanced the antitumour immune response to cell-based, granulocyte-macrophage colony-stimulating factor-secreting vaccines in mice.^23^ These results indicate that chemotherapy may be interesting to combine with immunotherapy, but the dosage and timing of chemotherapy might be critical to the effects of the combinatory treatment.^24^

The limitations of the study include open design and absence of a control group; however, at the time when the study was started, no standard treatment was available, and given the clinical status of the patients (stage IV melanoma), a placebo controlled group could not be justified.

In conclusion, in this study in patients with MAGE-A3-positive metastatic cutaneous melanoma, treatment with MAGE-A3 vaccination administered concurrently with dacarbazine was generally well tolerated and induced MAGE-A3-specific humoral response. This is one of the first studies including gene profile as a predictive marker. Although benefit remained much lower than what has been reported with checkpoint inhibitors since the time of this study, at the time when new trials are considering combinations of checkpoint inhibitors with chemotherapy or vaccination, and predictive markers are requested, the present results provide some interesting information.
