# Supplementary material for: Safety and immunogenicity of MAGE-A3 cancer immunotherapeutic with dacarbazine in patients with MAGE-A3-positive metastatic cutaneous melanoma: an open phase I/II study with a first assessment of a predictive gene signature
Source: ESMO Open. 2017 Nov 14;2(5):e000203. doi: 10.1136/esmoopen-2017-000203 (PMC5687540; doi:10.1136/esmoopen-2017-000203)
Supplement: Supplementary file 1 [file esmoopen-2017-000203supp001.pdf]

Supplementary materials

1. Details of inclusion/exclusion criteria

Patients with serum lactate dehydrogenase (LDH) ≤1.5 times the upper normal limit, no involvement of the central nervous system and normal organ functions were eligible for the study.

Patients were excluded if they were scheduled to receive anticancer therapies other than those specified in the protocol (including but not limited to [bio-]chemotherapeutic, immunomodulating agents and radiotherapy); the following therapies were allowed: limb perfusion performed at least 4 weeks prior to first study treatment administration, cancer immunotherapeutics containing a tumor antigen other than MAGE-A3 (provided the last administration took place at least 8 weeks before the first administration of study treatment). Patients were excluded if they had previous or concomitant malignancies at other sites, except effectively treated malignancy that was considered by the investigator highly likely to have been cured. Patients with a history of allergic disease or reactions likely to be exacerbated by any component of the study product, with autoimmune disease (with the exception of vitiligo), a family history of congenital or hereditary immunodeficiency, concurrent severe medical problems unrelated to the malignancy, or psychiatric or addictive disorders were ineligible. Patients with uncontrolled bleeding disorders and HIV-positive patients were also excluded.

2. Autoimmune diseases

Autoimmune diseases and other immune-mediated inflammatory disorders to be reported were:

- Neuroinflammatory diseases (e.g. optic neuritis, multiple sclerosis, demyelinating disease, transverse myelitis, encephalitis, Guillain-Barré syndrome, myasthenia gravis);

- Musculoskeletal disorders (e.g. systemic lupus erythematosus, Sjogren’s syndrome, scleroderma, rheumatoid arthritis, juvenile rheumatoid arthritis, inflammatory rheumatic arthritis, spondylarthropathies like reactive arthritis and ankylosing spondylitis);
- Gastrointestinal disorders (e.g. inflammatory bowel disease, Crohn’s disease, ulcerative colitis, ulcerative proctitis, coeliac disease);
- Thyroid diseases (e.g. Grave's disease [hyperthyroiditis], Hashimoto thyroiditis [hypothyroiditis], autoimmune thyroiditis);
- Skin disorders (e.g. cutaneous lupus, dermatomyositis, vitiligo, erythema nodosum, psoriasis, psoriatic arthropathy, Stevens-Johnson syndrome, Raynaud's phenomenon);
- Other diseases/disorders (e.g. autoimmune hemolytic anemia, antiphospholipid syndrome, insulin-dependent diabetes mellitus, idiopathic thrombocytopenic purpura, autoimmune hepatitis, autoimmune glomerulonephritis, autoimmune uveitis, sarcoidosis, Addison’s disease, vasculitis).

3. Assessment of clinical response

Response criteria were based on a set of measurable lesions identified at baseline as target lesions (i.e. ≥20 mm), and followed-up until disease progression. All measurable lesions up to a maximum of five lesions per organ and 10 lesions in total, representative of all involved organs, were identified as target lesions and were measured at baseline. Target lesions were selected on the basis of their size (those with the longest diameter [LD]) and their suitability for accurate repetitive measurements (either by imaging techniques or clinically). A sum of LD for all target lesions was calculated and reported as the baseline sum LD, and was used as reference to characterize the objective tumor response. All other lesions (or sites of disease) were identified as non-target lesions, and were also recorded and measured at baseline. Measurements were not required, but the presence or absence of each was recorded throughout follow-up.

Complete response (CR) was defined as disappearance of all target lesions; partial response (PR) (for patients with target lesions ≥20 mm) was defined at least a 30% decrease in the sum of the LD of target lesions taking as reference the baseline sum LD; stable disease (SD) was defined as neither sufficient shrinkage to qualify for PR (for patients with target lesions ≥20 mm) or CR (for patients with target lesions <20 mm), nor sufficient increase to qualify for progressive disease (PD) taking as references the smallest sum LD since the treatment started; PD was defined as, for patients with target lesions ≥20 mm, at least a 20% increase in the sum of LD of target lesions taking as references the smallest sum LD recorded since the treatment started or the appearance of one or more new lesions, or both of these; or, for patients with target lesions <20 mm, clear increase of diameters of target lesions taking as references the smallest diameters recorded since the treatment started or the appearance of one or more new target lesions, or both of these (according to investigator).

Mixed responses (MR) were not taken into account in the definition of a response for the secondary endpoint as all MR are either a SD or a PD. Indeed, regression of several target lesions but with the appearance of one new lesion was considered as a PD. Such MR could be explained by an adequate biological activity of the study treatment but with a resistance of the new tumor lesion to the specific immune response (i.e. HLA Class I loss, MAGE-A3-negative metastasis). Therefore, this recorded information was used for the descriptive assessment of the biological activity of the study treatment (secondary endpoint).

For patients who presented with objective tumor responses according to the Response Evaluation Criteria In Solid Tumors (RECIST) criteria, MR was defined as at least 30% decrease in the LD occurring in at least one target lesion recorded and measured at baseline. Such response occurring in patients with SD or PD status of the target lesions and without the appearance of new lesions was classified as “SD with target lesion regression” or “PD with target lesion regression”, respectively. For patients with disease non-evaluable according to the

RECIST criteria, MR was defined as clear decrease of diameters occurring in at least one target lesion recorded and measured at baseline. In patients with SD or PR status of the LD of target lesions, MR was defined as an appearance of one or more new lesions and was therefore classified as “SD with new lesion” or “PR with new lesion”.

For evaluation of non-target lesions, CR was defined as disappearance of all non-target lesions and normalization of tumor marker level; incomplete response (PR)/SD was defined as persistence of one or more non-target lesion(s); and PD was defined as the appearance of one or more new lesions and/or unequivocal progression of existing non-target lesions.

Slow progressive disease (SPD) was defined as the occurrence of all of the following criteria: World Health Organization performance status of 0 or 1, LDH ≤3 times the normal upper limit, no appearance of visceral metastases other than in the lung, and not meeting any criteria for permanent stopping of study treatment. SPD status was not considered as a “clinical success event” of this study and was not an endpoint of the study, but corresponded to specific protocol conventions that allowed continuing investigational treatment even in case of PD. This progression was not considered as a withdrawal criterion.

Patients with a global deterioration of their health status, requiring discontinuation of treatment without objective evidence of disease progression at that time, were reported as “symptomatic deterioration”. Every effort was made to document the objective progression even after discontinuation of treatment. Whenever it was difficult to distinguish residual disease from normal tissue, it was recommended that the residual lesion was investigated (fine needle aspirate/biopsy) before confirming the response status.

Progression-free survival (PFS) and PFS after initial slow progressive disease (SPD) was defined as the time from first dose to either the date of PD (PD or SPD for PFS or PD for PFS after initial SPD) or the date of death (regardless of the reason), whichever occurred first.

Patients who were still alive at the time of analysis and without any documented disease progression were censored at the date of their last tumor assessment.

Overall survival was defined as the time from first dose to the date of death (regardless of the reason). Patients still alive at the time of this analysis were censored at the date of “last known to be alive”.

Supplementary Table S1. Study procedures

		Cycle 1												
Visit no.	***Screening***	***1***	***2***	***3***	***4***	***5***	***6***	***7***	***8***	***9***	***10***	***11***	***12***	***13***
Beginning of week no.	-	1	4	7	10	13	16	19	22	25	28	31	34	37
**MAGE-A3 immunotherapeutic administration no.**	**-**	**1**	**2**	**3**	**4**	**5**	**6**	**7**	**8**	**9**	**10**	**11**	**12**	**-**
**Chemotherapy course no.**	**-**	**1**	**2**	**3**	**4**	**5**	**6**	**7**	**8**					
Informed consent	●													
Tumor biopsy for MAGE-A3 expression	●													
Check inclusion/exclusion criteria	●	●												
Medical history	●													
**Clinical evaluations**														
Physical examination and recording of ECOG performance status	●	●	●	●	●	●	●	●	●	●	●	●	●	●
Tumor imaging	●					●				●				●
Tumor response evaluation						●				●				●
**Safety assessments**														
Recording of SAEs	●[1]	●	●	●	●	●	●	●	●	●	●	●	●	●
Autoimmune diseases recorded		●	●	●	●	●	●	●	●	●	●	●	●	●
**Laboratory assessments**														
Urine sampling for:														
Urine pregnancy test	●	●	●	●	●	●	●	●	●	●	●	●	●	
Urine chemistry	●					●				●				●
Blood sampling[2] for:														
Hematological tests	●	●				●				●				●
Blood chemistry	●	●				●				●				●
Coagulation tests	●	●				●				●				●
Autoimmunity tests/TSH	●					●				●				●
Whole blood sampling (150 ml) for PBMC collection for cellular immunity tests		●				●				●				●
Check criteria for postponement or permanent stopping of study treatments			●	●	●	●	●	●	●	●	●	●	●	
Recording of concomitant medication		●	●	●	●	●	●	●	●	●	●	●	●	●

			Cycle 2		
Visit no.	***14***	***15***	***16***	***17***	***18***
Beginning of week no.	38	44	50	56	59
**MAGE-A3 immunotherapeutic administration no.**	**13**	**14**	**15**	**16**	-
**Clinical evaluations**					

Physical examination and recording of ECOG performance status	●	●	●	●	●
Tumor imaging					●
Tumor response evaluation					●
**Safety assessments**					
Recording of SAEs	●	●	●	●	●
Autoimmune diseases recorded	●	●	●	●	●
**Laboratory assessments**					
Urine sampling for:					
Urine pregnancy test	●	●	●	●	
Urine chemistry	●	●	●		●
Blood sampling[2] for:					
Hematological tests	●	●	●		●
Blood chemistry	●	●	●		●
Coagulation tests	●	●	●		●
Autoimmunity tests/TSH					●
Whole blood sampling (150 ml) for PBMC collection for cellular immunity tests					●
Check criteria for postponement or permanent stopping of study treatment	●	●	●	●	
Recording of concomitant medication	●	●	●	●	●

			**Cycle 3**		
Visit no.	***19***	***20***	***21***	***22***	***23***
Date of Visit	V17 + 3 months	V19 + 3 months	V20 + 3 months	V21 + 3 months	V22 + 3 weeks
**MAGE-A3 immunotherapeutic administration no.**	**17**	**18**	**19**	**20**	**-**
**Clinical evaluations**					
Physical examination and recording of ECOG performance status	●	●	●	●	●
Tumor imaging		●		●	
Tumor response evaluation		●		●	
**Safety assessments**					
Recording of SAEs	●	●	●	●	●
Autoimmune diseases recorded	●	●	●	●	●
**Laboratory assessments**					
Urine sampling for:					

Urine pregnancy test	●	●	●	●	
Urine chemistry		●		●	
Blood sampling[2] for:					
Hematological tests	●	●	●	●	
Blood chemistry		●		●	
Coagulation tests		●		●	
Autoimmunity tests/TSH		●		●	
Whole blood sampling (150 ml) for PBMC collection for cellular immunity tests					●
Check criteria for postponement or permanent stopping of study treatment	●	●	●	●	
Recording of concomitant medication	●	●	●	●	●

	Cycle 4					
Visit no.	***24***	***25***	***26***	***27***	***28***	***29***
Date of Visit	V22 + 6 months	V24 + 6 months	V25 + 3 weeks	V25 + 6 months	V27 + 6 months	V28 + 30 days
**MAGE-A3 immunotherapeutic administration no.**	**21**	**22**	-	**23**	**24**	**Concluding Visit**
**Clinical evaluations**						
Physical examination and recording of ECOG performance status	●	●	●	●	●	●
Tumor imaging	●	●		●		●
Tumor response evaluation	●	●		●		●
**Safety assessments**						
Recording of SAEs	●	●	●	●	●	●
Autoimmune diseases recorded	●	●	●	●	●	●
**Laboratory assessments**						
Urine sampling for:						
Urine pregnancy test	●	●		●	●	
Urine chemistry	●	●		●	●	●
Blood sampling[2] for:						
Hematological test	●	●		●	●	●
Blood chemistry	●	●		●	●	●
Coagulation tests	●	●		●	●	●
Autoimmunity tests/TSH	●	●		●	●	●
Whole blood sampling (150 ml) for PBMC collection for cellular immunity tests			●			●

Check criteria for postponement or permanent stopping of study treatment	●	●		●	●	
Recording of concomitant medication	●	●	●	●	●	●
Study conclusion						●

No., number; ECOG, Eastern Cooperative Oncology Group performance status; SAEs, serious adverse events; TSH, thyroid stimulating hormone; PBMC, peripheral blood mononuclear cell

[1]During screening only SAEs related to study participation or GSK concomitant medication were recorded

[2]Blood sampling for assessment of MAGE-A3-specific antibody response is indicated in Figure 1 (Study design) in the main manuscript

Supplementary Table S2. Demographic and disease characteristics of the study patients (Total treated population; N=48)

Characteristics	Value or n
**Age (years)**, mean (SD)	55.4 (16.0)
**Sex**	
Female, n (%)	21 (43.8)
Male, n (%)	27 (56.3)
**Time since initial disease diagnosis (years)**	
≤1 year, n (%)	9 (18.8)
1–3 years, n (%)	21 (43.8)
3–5 years, n (%)	7 (14.6)
5–10 years, n (%)	8 (16.7)
>10 years, n (%)	2 (4.2)
unknown, n (%)	1 (2.1)
**Primary tumor status**	
Unresected, n (%)	5 (10.4)
Not recurrent, resected, no residual tumor, n (%)	34 (70.8)
Not recurrent, resected, residual tumor, n (%)	4 (8.3)
Recurrent, n (%)	4 (8.3)
Missing, n (%)	1 (2.1)
**Regional lymph node dissection**	
yes, n (%)	29 (60.4)
no, n (%)	19 (39.6)
**Prior adjuvant treatment**	
yes, n (%)	18 (37.5)
no, n (%)	30 (62.5)
**Prior surgery**	
yes, n (%)	44 (91.7)
no, n (%)	4 (8.3)

N, total number of patients; SD, standard deviation; n (%), number (percentage) of patients in a given category

Supplementary Figure S1. Progression-free survival (PFS) (**A**) and PFS after initial slow progressive disease (SPD) (**B**) by gene signature (Total treated population)

A.

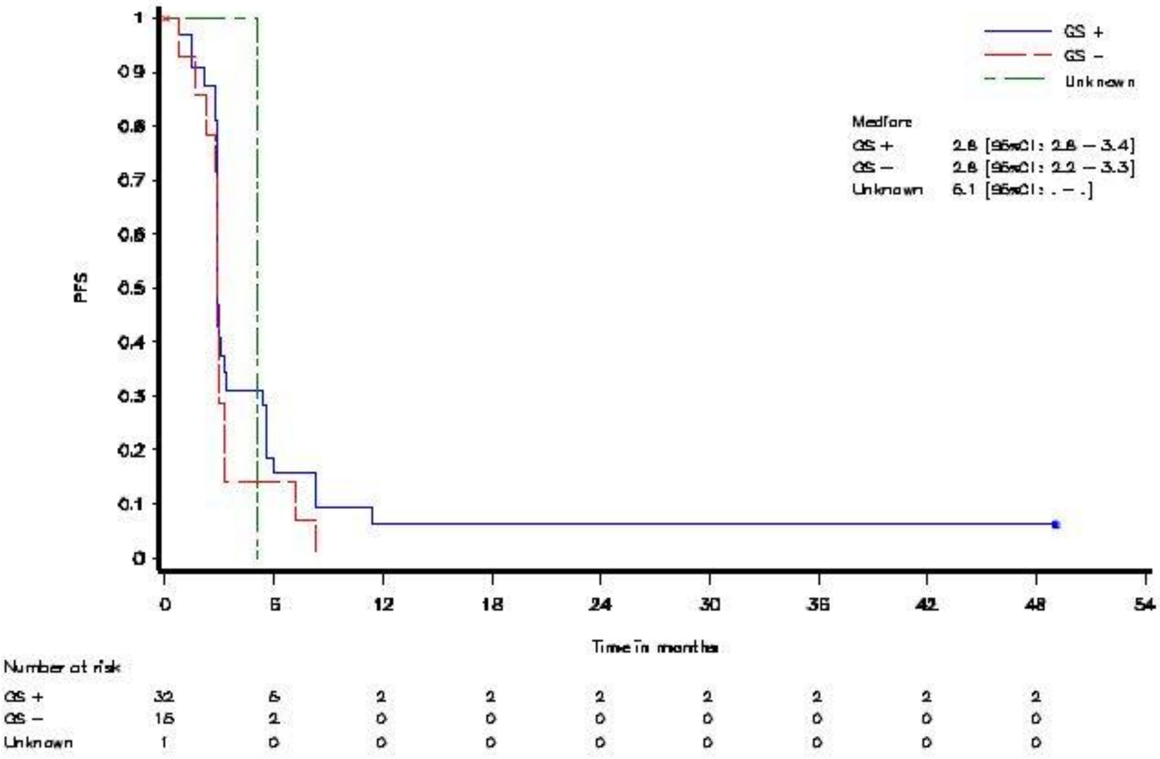

B.

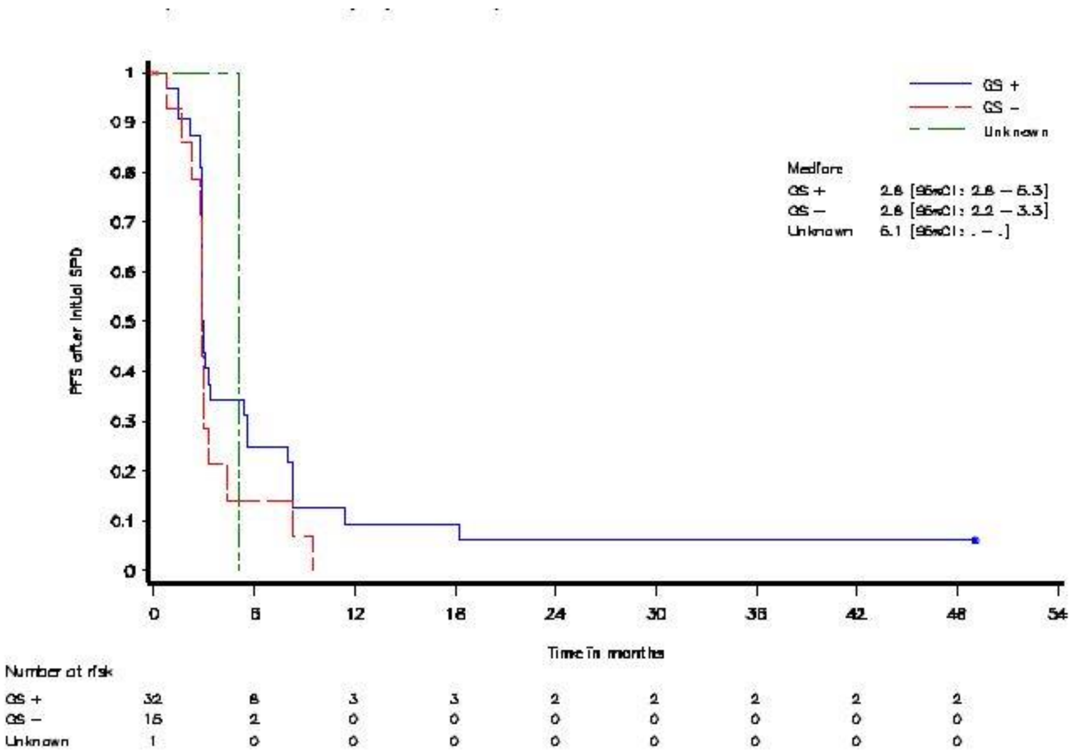

GS+, patients presenting gene signature; GS-, patients without gene signature; CI, confidence interval

Supplementary Figure S2. Follow-up duration by gene signature (Total treated population)

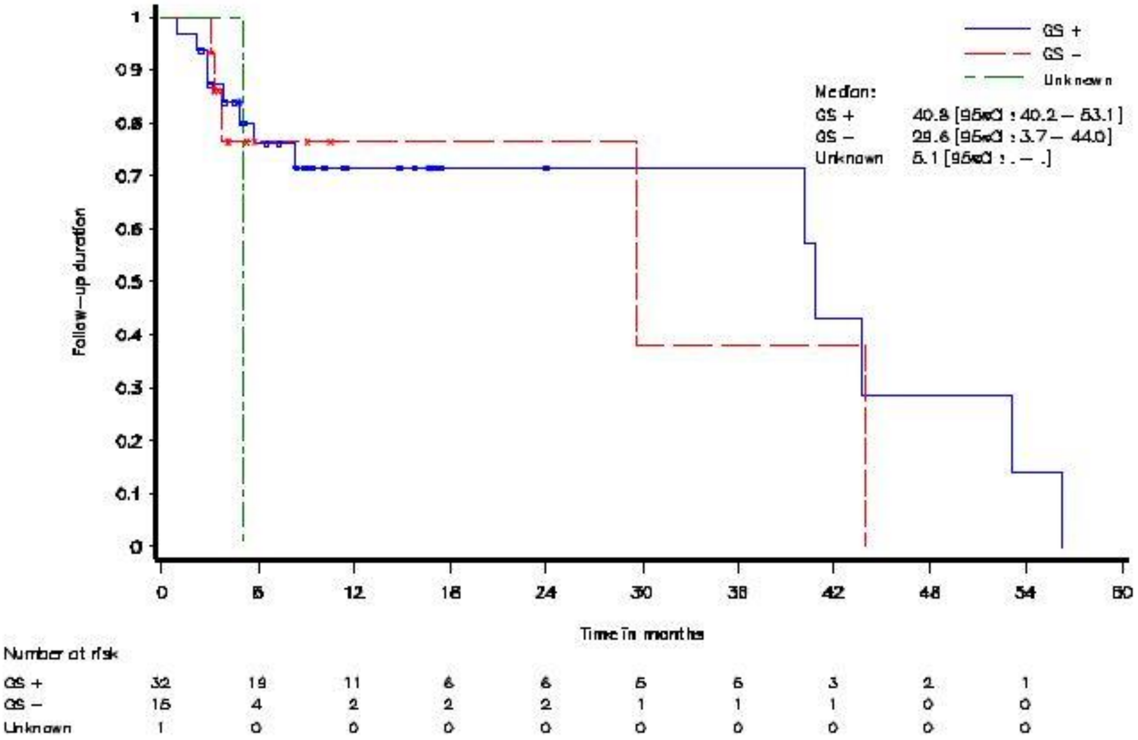

GS+, patients presenting gene signature; GS-, patients without gene signature; CI, confidence interval
